# Potent antiplasmodial extracts and fractions from *Terminalia mantaly* and *Terminalia superba*

**DOI:** 10.1186/s12936-018-2298-1

**Published:** 2018-04-03

**Authors:** Cedric D. J. Mbouna, Rufin M. T. Kouipou, Rodrigue Keumoe, Lauve R. Y. Tchokouaha, Patrick V. T. Fokou, Brice M. T. Tali, Dinkar Sahal, Fabrice F. Boyom

**Affiliations:** 1 Antimicrobial and Biocontrol Agents Unit, Laboratory for Phytobiochemistry and Medicinal Plants Studies, Faculty of Science, University of Yaoundé I, P.O. Box 812, Yaoundé, Cameroon; 2 Institute for Medical Research and Medicinal Plants Studies (IMPM), Yaoundé, P.O. Box 6163, Yaoundé, Cameroon; 3 Malaria Research Laboratory, International Centre for Genetic Engineering and Biotechnology, Aruna Asaf Ali Marg, New Delhi, 110067 India

**Keywords:** *Plasmodium falciparum*, Antiplasmodial, Selectivity, *Terminalia mantaly*, *Terminalia superba*

## Abstract

**Background:**

The emergence and spread of malaria parasites resistant to artemisinin-based combination therapy stresses the need for novel drugs against malaria. Investigating plants used in traditional medicine to treat malaria remains a credible option for new anti-malarial drug development. This study was aimed at investigating the antiplasmodial activity and selectivity of extracts and fractions from *Terminalia mantaly* and *Terminalia superba* (Combretaceae) that are used in Cameroon to treat malaria.

**Methods:**

Twelve methanolic (m) and water (w) extracts obtained by maceration of powdered dried leaves (l), stem bark (sb) and root (r) of *Terminalia mantaly* (*Tm*) and *Terminalia superba* (*Ts*) and 12 derived fractions of hexane, chloroform, ethyl acetate and 4 final residues of selected extracts were assessed for antiplasmodial potential in vitro against the chloroquine-resistant *Pf*INDO and the chloroquine-sensitive *Pf*3D7 strains of *Plasmodium falciparum* using the SYBR green I-based fluorescence assay. The cytotoxicity of potent extracts and fractions was evaluated in vitro using the MTT assay on HEK239T cell line.

**Results:**

The antiplasmodial IC_50_ of extracts from both plants ranged from 0.26 to > 25 µg/mL. Apart from the extracts Tmrm and Tsrw that exerted moderate antiplasmodial activities (IC_50_: 5–20 µg/mL) and Tmrw that was found to be non-active at the tested concentrations (IC_50_ > 25 µg/mL), all other tested crude extracts exhibited potent activities with IC_50_ < 5 µg/mL. The aqueous extracts from the stem bark of *Terminalia mantaly* (Tmsbw) and the leaf of *Terminalia superba* (Tslw) displayed the highest antiplasmodial activities (IC_50_: 0.26–1.26 µg/mL) and selectivity (SI > 158) on both resistant PfINDO and sensitive Pf3D7 strains. Four fractions upon further extraction with chloroform and ethyl acetate (TmlwChl, TmsbwChl, TmsbwEA, TsrmEA) afforded from three selected crude extracts (Tmlw, Tmsbw, Tsrm) exhibited highly potent activities against both *P. falciparum* strains (IC_50_ < 2 µg/mL) and high selectivity (SI > 109).

**Conclusions:**

The results achieved in this work validate the reported traditional use of *Terminalia mantaly* and *Terminalia superba* to treat malaria. Moreover, the highly potent and selective fractions warrant further investigation to characterize the active antiplasmodial principles and progress them to rodent malaria models studies if activity and selectivity are evidenced.

## Background

Malaria is the world’s most important protozoan disease. In 2015, an estimated 214 million malaria cases occurred worldwide with an approximated 438,000 deaths. Sub-Saharan Africa is the most affected region based on statistics, with 88% of the overall malaria cases and 90% of all deaths [1]. In Africa, malaria constitutes over 10% of overall disease burden, accounting for 40% of public health expenditure, 30–50% of in-patient hospital admissions and up to 50% of out-patient visits in endemic areas, and thus represents a major hindrance to the socio-economic development [2].

Cameroon is amongst the most affected countries where malaria is the first major cause of morbidity and mortality amongst the most vulnerable patient groups such as children under 5 years of age, pregnant women, people living with HIV/AIDS and the poor [3]. In 2014, the whole Cameroonian population was at risk of malaria with 71% living in high transmission areas. *Plasmodium falciparum* is the causative agent of all reported cases of malaria in Cameroon [1]. Overall, thousands of people continue to die from malaria each year and despite extensive efforts to control the disease, it remains a major public health threat [4, 5]. With persistent severe malarial morbidity and increasing resistance to malaria drugs, including the recently introduced, first-line, artemisinin-based combination therapy (ACT) [6, 7], there is a compelling need for new and improved treatments for malaria. History reveals that medicinal plants have always been an important source of chemotherapeutic agents, and indigenous healthcare systems have always played a vital role in the management of community health and discovery of novel chemotherapeutic agents against malaria. For example, two of the most important anti-malarial drugs, namely quinine and artemisinin, have their respective origins in the medicinal plants *Cinchona officinalis* and *Artemisia annua* [8]. In Cameroon, pharmacopeia plants are widely used to treat malaria and several other diseases, particularly in remote areas where access to standard treatments is limited. *Terminalia mantaly* and *Terminalia superba* have been reported in such areas in Cameroon as sources of treatment for various diseases, including malaria and/or related symptoms [9]. However, little is known about the antiplasmodial activity of extracts from these plants. This study was therefore designed to investigate the antiplasmodial potential and cytotoxicity of extracts and fractions from leaves, stem back and roots of *Terminalia mantaly* and *Terminalia superba*.

## Methods

### Plants collection, extraction and fractionation

Leaf, stem bark and root samples were collected from *Terminalia mantaly* and *Terminalia superba* during August and September 2014 in Nkolbisson and Ngoa-Ekelle, Yaoundé, Cameroon. Plants were identified at the National Herbarium of Cameroon, Yaoundé where voucher specimens were deposited under the reference numbers 64212/HNC [*Terminalia mantaly* (*Tm*)] and 55030/HNC [*Terminalia superba* (*Ts*)], respectively.

The plant samples were air-dried (2 kg dry weight) and ground into fine powder using an electric mill (Hammer Mill, Leabon 9FQ, Zhengzhou, PRC). One kg of each plant part powder was separately macerated in 10 L of distilled water and methanol, respectively, for 72 h at room temperature (27–29 °C). The methanol macerates were filtered and filtrates evaporated using a rotary evaporator (Rotavapor, BUCHI 071, Switzerland) at 65 °C. The aqueous extracts were lyophilized at the Laboratory of Phytochemistry, Institute for Medical Research and Medicinal Plants Studies (IMPM), Yaoundé, Cameroon using a Virtis Wizard 2.0 Freeze Dryer Lyophilizer: Model: XLS-70. The dried crude extracts were subsequently subjected to antiplasmodial screening in vitro against the resistant *Pf*INDO and sensitive *Pf*3D7 strains. Crude extracts with potent antiplasmodial activity and high selectivity index were selected and subjected to successive solid–liquid solvent extractions. Cytotoxicity of extracts and fractions was assessed on HEK239T cells using MTT assay [10].

**Fig. 1:**
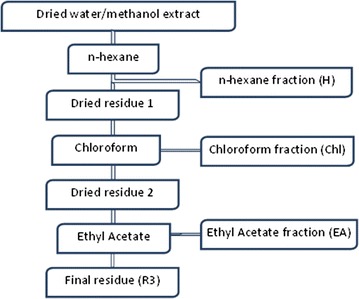
Scheme showing the successive solid–liquid extractions of the crude extracts using organic solvents

Dried aqueous and methanol crude extracts (100 g of *Tm*l^w^, *Tm*sb^w^, and *Ts*l^w^, and 20 g *Ts*r^m^) selected based on their antiplasmodial activity and selectivity (IC_50_ < 3 µg/mL; SI > 75 on both strains) were successively extracted with *n*-hexane (1 L), chloroform (1 L), and ethyl acetate (1 L) to afford *n*-hexane (H), chloroform (Chl), and ethyl acetate (EA) fractions, respectively, together with insoluble final residues (R3) as described in Fig. 1. The afforded fractions were filtered separately through Whatman No. 4 filter paper and were then concentrated using rotary evaporator. The dried fractions and final residues were weighed and the yields calculated relative to the weight of the starting crude extracts.

### In vitro cultivation of *Plasmodium falciparum*

Chloroquine-sensitive 3D7 (*Pf*3D7) and resistant INDO (*Pf*INDO) strains of *P. falciparum* were maintained at the Malaria Research Laboratory, International Centre for Genetic Engineering and Biotechnology, New Delhi, India, and used for in vitro blood stage testing of antiplasmodial activity of the plant extracts and fractions. *Plasmodium falciparum* culture was maintained according to the method described by Trager and Jensen [11] with minor modifications [12], in fresh O positive human erythrocytes suspended at 4% haematocrit in RPMI 1640 16.2 g/L (Sigma-Aldrich, New Delhi, India) containing 25 mM HEPES, 11.11 mM glucose, 0.2% sodium bicarbonate (Sigma-Aldrich, New Delhi, India), 0.5% albumax I (Gibco, Waltham, MA USA), 45 mg/L hypoxanthine (Sigma-Aldrich, New Delhi, India), and 50 mg/L gentamicin (Gibco, Waltham, MA USA) and incubated at 37 °C under a gas mixture of 5% O_2_, 5% CO_2_ and 90% N_2_. Every day infected erythrocytes were transferred into fresh complete medium to propagate the culture. At parasitaemia > 10%, the cultures were diluted into healthy red blood cells to reduce  % parasitaemia to 1–2% so as to maintain the cultures in stress free conditions.

### Dilution of crude extracts, fractions and positive control

Stock solutions of plant extracts and fractions were prepared at 1 mg/mL in dimethyl sulfoxide (DMSO) while chloroquine (Sigma-Aldrich, New Delhi, India) stock solution used as standard drug was prepared in water (Milli-Q grade, The Netherlands) at 1 mM. All stocks solutions were then diluted in 96-well, round-bottom, tissue culture-grade plates (Corning, USA) with fresh RPMI 1640 culture medium to achieve the required concentrations for testing. In all cases, except for chloroquine (positive control), the final solution contained 0.4% DMSO, which was found to be non-toxic to the parasite. Extracts and fractions were tested at concentrations ranging from 0.10 to 25 μg/mL, and chloroquine at 1 µM highest concentration.

### In vitro antiplasmodial assays

Crude extracts and fractions were evaluated for antiplasmodial activity against *P. falciparum* INDO and 3D7 strains. For drug screening, the SYBR green I-based fluorescence assay was set up as described by Smilkstein et al. [13]. Precisely, 100 μL of Sorbitol-synchronized parasites [14] were incubated under normal culture conditions (37 °C, 5% CO_2_, 5% O_2_, 90% N_2_) at 1% parasitaemia and 2% haematocrit in flat-bottomed, 96-well plates (Corning, USA) in the absence or presence of increasing concentrations of crude extracts or fractions for 48 h. Chloroquine (Sigma-Aldrich, New Delhi, India) was used as positive control, while 0.4% (v/v) DMSO was used as the negative control. Upon incubation, 100 μL of SYBR green I lysis buffer (Tris (20 mM; pH 7.5), EDTA (5 mM), saponin (0.008%, w/v), and Triton X-100 (0.08%, v/v))was added to each well and mixed gently twice, then incubated in dark at 37 °C for 1 h. Fluorescence was then measured with a Victor fluorescence multiwell plate reader (Perkin Elmer, Waltham, MA, USA) with excitation and emission wavelength bands centred at 485 and 530 nm, respectively. The fluorescence counts were plotted against drug concentration and the 50% inhibitory concentration (IC_50_) was determined by analysis of dose–response curves using non-linear regression. Resistance indexes were calculated as RI = IC_50_ *Pf*INDO/IC_50_*Pf*3D7. Results were validated microscopically by examination of Giemsa-stained smears of extract-treated/untreated parasite cultures.

### Assessment of the cytotoxicity of active extracts and fractions on HEK239T cells

The cytotoxic effect of antiplasmodial extracts and fractions was assessed using the MTT assay [10], targeting HEK239T cells cultured in complete medium containing 13.5 g/L DMEM (Gibco, Waltham, MA USA), 10% fetal bovine serum (Gibco, Waltham, MA USA), 0.21% sodium bicarbonate (Sigma-Aldrich, New Delhi, India) and 50 μg/mL gentamicin (Gibco, Waltham, MA USA). Essentially, HEK239T cells at 5 × 10^3^ cells/200 μL/well were seeded into 96-well, flat-bottomed, tissue culture plates (Corning, USA) in complete medium. Fifty µL of serially diluted extracts and fractions solutions (≤ 200 µg/mL) were added after 24 h of seeding and then incubated for 48 h in a humidified atmosphere at 37 °C and 5% CO_2_. DMSO was added as positive inhibitor at 10% v/v. Twenty µL of a stock solution of MTT (5 mg/mL in 1× phosphate-buffered saline) were added to each well, gently mixed and incubated for an additional 4 h. After spinning the plate at 1500 rpm for 5 min, the supernatant was carefully removed and 100 μL of DMSO (stop agent) was added. Formazan formation was read on a microtiter plate reader (Versa Max Microplate Reader, Molecular Devices, USA) at 570 nm. The 50% cytotoxic concentrations (CC_50_) of extracts and fractions were determined by analysis of dose–response curves. Selectivity indices (CC_50_/IC_50_) were calculated for each extract and fraction.

## Results

The present study was designed to assess the biological properties of *Terminalia mantaly* and *Terminalia superba* as sources of antiplasmodial agents with potential to be further investigated for anti-malarial drug development. The results achieved are summarized and discussed below.

### Antiplasmodial activity and cytotoxicity of crude extracts from *Terminalia mantaly* and *Terminalia superba*

**Fig. 2:**
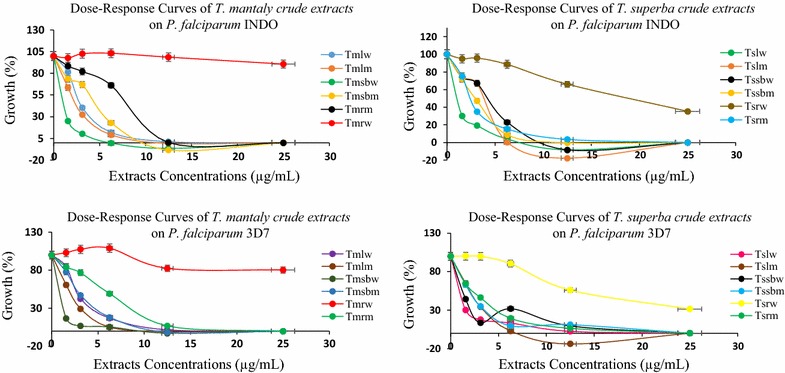
Dose–response curves of *Terminalia mantaly* and *Terminalia superba* crude extracts on *Plasmodium falciparum* INDO and 3D7 strains (*Tm: Terminalia mantaly*; *Ts: Terminalia superba*; l: leaf; sb: stem bark; r: roots; w: water; m: methanol)

A total of 12 crude extracts were prepared from the leaves, stem bark and roots of *Terminalia mantaly* and *Terminalia superba* by maceration in distilled water and methanol and further assessed for biological activities. Table 1 summarizes the results of crude extracts preparation and biological screening against *P. falciparum* INDO and 3D7 strains and HEK239T mammalian cells. The dose–response curves schematizing the activity of crude extracts are presented in Fig. 2.

**Table 1:** Crude extracts preparation yields and activity parameters

Plant	Part	Solvent	Extract code	Extraction yield (%)	IC_50_ ± SD (µg/mL)		RI	CC_50_ (µg/mL)	Selectivity Index	
					*Pf*INDO	*Pf*3D7			*Pf*INDO	*Pf*3D7
*T. mantaly*	Leaf	Water	*Tm*l^w^	35.50	2.09 ± 0.06	2.66 ± 0.31	0.79	> 200	> 95.69	> 75.19
		Methanol	*Tm*l^m^	19.54	2.69 ± 0.04	2.61 ± 0.43	1.03	> 200	> 74.35	> 76.63
	Stem bark	Water	*Tm*sb^w^	19.93	0.26 ± 0.02	1.03 ± 0.04	0.25	> 200	> 769.23	> 194.17
		Methanol	*Tm*sb^m^	19.32	3.63 ± 0.50	2.91 ± 0.12	1.25	> 200	> 55.10	> 68.73
	Root	Water	*Tm*r^w^	07.45	> 25	> 25	–	–	–	–
		Methanol	*Tm*r^m^	09.01	7.01 ± 0.82	5.04 ± 0.68	1.39	> 200	> 28.53	> 39.68
*T. superba*	Leaf	Water	*Ts*l^w^	17.18	0.57 ± 0.06	1.26 ± 0.12	0.45	> 200	> 350.88	> 158.73
		Methanol	*Ts*l^m^	12.07	3.38 ± 0.54	2.13 ± 0.13	1.59	> 200	> 59.17	> 93.90
	Stem bark	Water	*Ts*sb^w^	14.15	3.70 ± 0.03	1.42 ± 0.09	2.61	> 200	> 54.05	> 140.85
		Methanol	*Ts*sb^m^	10.35	2.68 ± 0.20	1.85 ± 0.13	1.44	> 200	> 74.63	> 108.11
	Root	Water	*Ts*r^w^	04.54	18.88 ± 0.14	16.43 ± 0.14	1.15	–	–	–
		Methanol	*Ts*r^m^	02.27	2.38 ± 0.08	2.28 ± 0.20	1.04	> 200	> 84.03	> 87.72
Reference drug		Chloroquine (µM)		–	0.40 ± 0.00	0.04 ± 0.01	10.00	–	–	–

Activity data are presented as means of triplicate experiments

IC_50_: 50% inhibitory concentration; CC_50_: 50% cell cytotoxic concentration, cytotoxicity was tested against HEK239T cells; SD: standard deviation from triplicate experiments; RI: resistance index; resistance index was calculated as the ratio of IC_50_ *Pf*INDO_resistant_/IC50*Pf*3D7_sensitive_; SI: selectivity index; *Tm*: *Terminalia mantaly*; *Ts*: *Terminalia superba*; l: leaf; sb: stem bark; r: root; w: water; m: methanol

Results from Table 1 show that plant extraction yields varied from 7.45 to 35.50% for *Terminalia mantaly* parts and from 2.27 to 17.18% for *Terminalia superba* parts. Yields were dependent upon plant species, parts and solvent of extraction. In general, leaves as source and water as extractant gave higher yields than was the case with methanol. Water is environment-friendly, making it an ideal and cheap solvent for the extraction of bioactive and safe principles from any source.

The antiplasmodial activity (expressed as IC_50_) of crude extracts ranged from 0.26 to > 25 µg/mL with nine extracts showing very promising potency (IC_50_ < 5 µg/mL). The aqueous extract from *Terminalia mantaly* stem bark (*Tm*sb^w^) showed the best antiplasmodial activity and selectivity, respectively, on *Pf*INDO and *Pf*3D7 strains with IC_50_ values of 0.26 µg/mL (SI_*Pf*INDO_ > 769.23) and 1.03 µg/mL (SI_*Pf*3D7_ > 194.17). For *Terminalia superba*, the aqueous extract from leaves (*Ts*l^w^) also showed high antiplasmodial activity and selectivity with IC_50_ values of 0.57 µg/mL (SI_*Pf*INDO_ > 350.88) and 1.26 µg/mL (SI_*Pf*3D7_ > 158.73) on *Pf*INDO and *Pf*3D7 strains, respectively.

With most values hovering around one, resistance index data on crude samples (Table 1) suggests that the antiplasmodial metabolites present in the plant extracts may be equipotent against both chloroquine-sensitive and -resistant strains of *P. falciparum*. However, *Tm*sb^w^ (RI: 0.25), and *Ts*l^w^ (RI: 0.45) were clearly three to fourfold more active against *Pf*INDO than *Pf*3D7, respectively, suggesting interesting possibilities of unique and novel drug targets in the resistant strain. The selectivity indices of crude extracts ranging from > 28 to > 769 µg/mL suggest that the crude extracts not only exhibit potent antiplasmodial potency but also possess great selectivity in their action.

### Antiplasmodial activity and cytotoxicity of fractions from the most promising extracts

Four crude extracts showing promising antiplasmodial activity profile were selected and fractionated via successive solid–liquid extractions using three different organic solvents (*n*-hexane, chloroform, ethyl acetate). These were the aqueous extracts from leaf and stem bark of *Terminalia mantaly* (*Tm*l^w^: IC_50_*Pf*INDO: 2.09 µg/mL, SI_*Pf*INDO_ > 95; IC_50_*Pf*3D7: 2.66 µg/mL, SI_*Pf*3D7_ > 75; and *Tm*sb^w^: IC_50_*Pf*INDO: 0.26 µg/mL, SI_*Pf*INDO_ > 769; IC_50_*Pf*3D7: 1.03 µg/mL, SI_*Pf*3D7_ > 194), and the aqueous and methanolic extracts from leaf and root of *Terminalia superba* (*Ts*l^w^: IC_50_*Pf*INDO: 0.57 µg/mL, SI_*Pf*INDO_ > 350; IC_50_*Pf*3D7: 1.26 µg/mL, SI_*Pf*3D7_ > 158; and *Ts*r^m^: IC_50_*Pf*INDO: 2.38 µg/mL, SI_*Pf*INDO_ > 84; IC_50_*Pf*3D7: 2.28 µg/mL, SI_*Pf*3D7_ > 87).

**Fig. 3:**
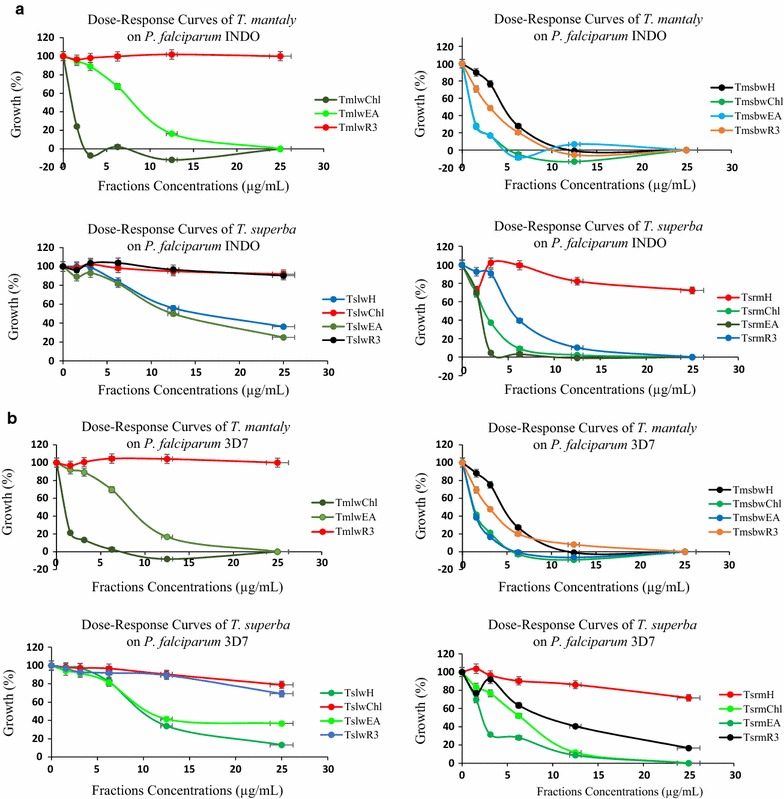
**a**, **b** Dose-response curves of fractions from *Terminalia mantaly* and *Terminalia superba* on *Plasmodium falciparum* INDO and 3D7 strains (*Tm*: *Terminalia mantaly*; *Ts*: *Terminalia superba*; *Tm*lw: *Terminalia mantaly* leaf water extract; *Tm*sbw: *Terminalia mantaly* stem bark water extract; *Ts*lw: *Terminalia superba* leaf water extract; *Ts*rm: *Terminalia superba* root methanol extract; H: hexane; Chl: chloroform; EA: ethyl acetate; R3: final residue)

Thus, this fractionation led to 3 fractions and 1 final residue for each crude extract, for a total of 12 fractions and 4 final residues that were tested for biological activities as reported in Table 2. The dose–response curves of the tested fractions against *P. falciparum* INDO and 3D7 are given in Fig. 3a, b.

**Table 2:** Yields of crude extracts fractionation and activity parameters

Plant	Crude extract	Solvent	Fraction code	Fraction yield (%)	IC_50_ ± SD (µg/mL)		RI	CC_50_ (µg/mL)	Selectivity index	
					*Pf*INDO	*Pf*3D7			*Pf*INDO	*Pf*3D7
*T. mantaly*	*Tm*l^w^	Hexane	*Tm*l^w^H	0	–	–	–	–	–	–
		Chl	*Tm*l^w^Chl	20.78	0.36 ± 0.01	0.96 ± 0.01	0.37	> 200	> 555.55	> 208.33
		EA	*Tm*l^w^EA	2.54	6.74 ± 0.49	6.88 ± 0.58	0.98	> 200	> 29.67	> 29.07
		–	*Tm*l^w^R3	43.79	> 25	> 25	–	–	–	–
	*Tm*sb^w^	Hexane	*Tm*sb^w^H	0.16	4.50 ± 0.24	4.40 ± 0.27	1.02	> 200	> 44.44	> 45.45
		Chl	*Tm*sb^w^Chl	0.67	0.56 ± 0.05	1.12 ± 0.07	0.5	> 200	> 357.14	> 178.57
		EA	*Tm*sb^w^EA	1.26	0.68 ± 0.14	1.35 ± 0.42	0.5	> 200	> 294.11	> 148.15
		–	*Tm*sb^w^R3	31.35	2.80 ± 0.21	2.44 ± 0.16	1.15	> 200	> 71.43	> 8.97
*T. superba*	*Ts*lw	Hexane	*Ts*l^w^H	0.7	14.09 ± 1.88	6.99 ± 0.68	2.02	126.03	> 14.19	> 28.61
		Chl	*Ts*l^w^Chl	4.74	> 25	> 25	–	–	–	–
		EA	*Ts*l^w^EA	4.47	6.89 ± 0.94	7.06 ± 0.76	0.97	> 200	> 29.03	> 28.33
		–	*Ts*l^w^R3	61.9	> 25	> 25	–	–	–	–
	*Ts*r^m^	Hexane	*Ts*r^m^H	19.36	> 25	> 25	–	–	–	–
		Chl	*Ts*r^m^Chl	5.54	2.26 ± 0.09	4.93 ± 0.73	0.46	> 200	> 88.49	> 40.57
		EA	*Ts*r^m^EA	6.18	1.82 ± 0.04	1.65 ± 0.24	1.1	> 200	> 109.89	> 121.21
		–	*Ts*r^m^R3	63.82	5.22 ± 0.26	4.70 ± 1.47	1.11	> 200	> 38.31	> 42.55
Reference drug		Chloroquine (µM)		–	0.40 ± 0.00	0.04 ± 0.00	10	–	–	–

Activity data are presented as means of triplicate experiments

IC_50_: 50% inhibitory concentration; CC_50_: 50% cell cytotoxic concentration, cytotoxicity was tested against HEK239T cells; SD: standard deviation from triplicate experiments; RI: resistance index; resistance index was calculated as the ratio of IC_50_ *Pf*INDO_resistant_/IC_50_*Pf*3D7_sensitive_; SI: selectivity index; *Tm: Terminalia mantaly*; *Ts*: *Terminalia superba*; *Tm*l^w^: *Terminalia mantaly* leaf water extract; *Tm*sb^w^: *Terminalia mantaly* stem bark water extract; *Ts*l^w^: *Terminalia superba* leaf water extract; *Ts*r^m^: *Terminalia superba* root methanol extract; R3: final residue; H: hexane; Chl: chloroform; EA: ethyl acetate

Overall, 11 fractions showed antiplasmodial potential against both resistant and sensitive strains of *P. falciparum,* with IC_50_ values ranging from 0.36 to 14.09 µg/mL. Four out of those fractions codified *Tm*l^w^Chl, *Tm*sb^w^Chl, *Tm*sb^w^EA, and *Ts*r^m^EA exhibited highly potent antiplasmodial activity with IC_50_ values ≤ 2 µg/mL on both strains, and high selectivity (SI > 109).

The chloroform fraction (*Tm*l^w^Chl) from the leaf aqueous extract of *Terminalia mantaly* (*Tm*l^w^) showed the highest antiplasmodial activity, with IC_50_*Pf*INDO = 0.36 µg/mL and IC_50_*Pf*3D7 = 0.96 µg/mL. Two fractions from the stem bark aqueous extract of *Terminalia mantaly* (*Tm*sb^w^) exhibited high activity mainly against the resistant *Pf*INDO strain, namely the chloroform fraction (*Tm*sb^w^Chl, IC_50_*Pf*INDO: 0.56 µg/mL against IC_50_*Pf*3D7: 1.12 µg/mL), and the ethyl acetate fraction (*Tm*sb^w^EA, IC_50_*Pf*INDO: 0.68 µg/mL against IC_50_*Pf*3D7: 1.35 µg/mL). These two fractions were found to be about twofold more active against the resistant *Pf*INDO strain than the sensitive *Pf*3D7, and also showed very good selectivity with SI > 148. The ethyl acetate fraction (*Ts*r^m^EA) of the root methanolic extract of *Terminalia superba* was also quite promising (IC_50_*Pf*INDO: 1.82 µg/mL, IC_50_*Pf*3D7: 1.65 µg/mL, SI > 109). Three other fractions from both plants (*Terminalia mantaly* and *Terminalia superba*) showed significant activity profiles with IC_50_ values ranging (2.26–4.93 µg/mL), viz*. Tm*sb^w^H (IC_50_*Pf*INDO: 4.50, IC_50_*Pf*3D7: 4.40 µg/mL), *Tm*sb^w^R3 (IC_50_*Pf*INDO: 2.80, IC_50_*Pf*3D7: 2.44 µg/mL), and *Ts*r^m^Chl (IC_50_*Pf*INDO: 2.26, IC_50_*Pf*3D7: 4.93 µg/mL). However, these latter fractions showed moderate selectivity (8 < SI > 71) compared to the more potent ones.

Crude extract fractionation led to fractions and final residues with varied activities against *P. falciparum* parasites. The overall more potent fraction *Tm*l^w^Chl (IC_50_*Pf*INDO: 0.36 µg/mL and IC_50_*Pf*3D7: 0.96 µg/mL; SI > 208) with an average 4.3-fold activity magnification derived from the crude extract *Tm*l^w^ (IC_50_*Pf*INDO: 2.09 µg/mL and IC_50_*Pf*3D7: 2.66 µg/mL; SI > 75). Fractionation of *Ts*r^m^ led to no significant change in activity. Fractionation of *Tm*sb^w^ (IC_50_*Pf*INDO: 0.26 µg/mL and IC_50_*Pf*3D7: 1.03 µg/mL; SI > 194) slightly reduced the antiplasmodial potency (but led to promising and selective fractions—IC_50_*Pf*INDO: 0.39–4.50 µg/mL and IC_50_*Pf*3D7: 1.12–4.40 µg/mL), whereas fractionation of extract *Ts*l^w^ (IC_50_*Pf*INDO: 0.57 µg/mL and IC_50_*Pf*3D7: 1.26 µg/mL) negatively impacted its potency leading to only two moderately active fractions (*Ts*l^w^H and *Ts*l^w^EA-IC_50_: 6.89–14.09 µg/mL).

The thresholds for the in vitro antiplasmodial activity of the plant extracts/fractions were based on the classification according to Gessler et al. [15] where the promise of the extract is based on its potency: IC_50_ < 10 µg/mL (very good); 10–50 µg/mL (moderate) and > 50 µg/mL (low activity). Based on this classification, results from this study indicate that most of the tested extracts and fractions exerted very good activities against both sensitive and resistant strains of *P. falciparum.*

Very few reports are available in the literature on the activity of the studied plants against *P. falciparum*. Among those, Ngemenya et al. [16] previously reported the antiplasmodial activity (IC_50_ of 19.5 µg/mL) of methanolic extract from the leaf of *Terminalia superba* against the chloroquine-sensitive *P. falciparum* F32 strain. This value is about five to ninefold greater than that of similar extract tested in this study against *Pf*INDO and *Pf*3D7. This activity discrepancy might be explained by the difference in parasite strains and approaches used and the specific features of parasites relating to drug susceptibility. Indeed, there might be a relationship between in vitro adaptation to culture of *P. falciparum* and drug-resistant characteristic of the strain. There is also the possibility of the emergence of a drug-resistant sub-population or of changes in the metabolic pathways during the course of in vitro routine culture maintenance [17]. Likewise, Adewunmi et al. [18] investigated the activity of root and stem of *Terminalia superba* against *Trypanosoma congolense* IL 1180 and reported IC_50_ values of 56.1 µg/mL (root ethanolic extract), 91.73 and 55.26 µg/mL for stem hexane and ethanolic extracts. These findings further highlight the potential of *Terminalia superba* as a source of anti-protozoan principles.

The antiplasmodial activities of extracts, fractions and isolated compounds from many *Terminalia* species have been previously reported. However, this study is reporting for the first time the antiplasmodial activity of extracts from *Terminalia mantaly*. Moreover, it is the first report on the antiplasmodial activity of *Terminalia* species against *P. falciparum* chloroquine-resistant INDO strain.

Muganga et al. [19] reported the antiplasmodial activity of *Terminalia mollis* crude methanolic extract (IC_50_: 3.84 µg/mL), aqueous extract (IC_50_: 4.66 µg/mL), ethyl acetate fraction (IC_50_: 2.10 µg/mL), aqueous fraction (IC_50_: 19.72 µg/mL) and isolated ellagic acid (IC_50_: 0.17 µg/mL) against *P. falciparum* 3D7 strain. Mohd Abd Razak et al. [20] reported the antiplasmodial activity of *Terminalia catappa* aqueous extract (IC_50_: 4.28 µg/mL), methanolic extract (IC_50_: 5.19 µg/mL) and dichloromethane extract (IC_50_: 5.29 µg/mL) on *P. falciparum* K1. Abiodun et al. [21] also reported the antiplasmodial activity of hexane, ethyl acetate and methanolic extracts from *Terminalia catappa* on *P. falciparum* K1 (IC_50_: 10.10, 3.05 and 7.42 µg/mL, respectively) and *P. falciparum* NF54 (IC_50_: 21.93, 6.68 and 9.40 µg/mL, respectively). Sanon et al. [22] reported the antiplasmodial activity of *Terminalia avicennioides* aqueous, methanolic and dichloromethane extracts from leaf and stem bark with IC_50_ values ranging from 1.60 to 7.40 µg/mL on *P. falciparum* K1. Ouattara et al. [23] reported the activity of *Terminalia avicennioides* ethyl acetate and butanol crude extracts against *P. falciparum* K1, while ellagic acid isolated from the leaf showed potent antiplasmodial activity with an IC_50_ of 0.52 µM. Bavagan et al. [24] previously reported the antiplasmodial activity of *Terminalia chebula* hexane, ethyl acetate, acetone, and methanolic extracts on *P. falciparum* 3D7 with IC_50_ values of 51.91, 67.45, 4.76, 42.98 µg/mL, respectively. They equally highlighted the antiplasmodial potential of aqueous extracts from stem bark and stem wood of *Terminalia spinosa* on *P. falciparum* chloroquine-resistant ENT36 (IC_50_: 29.50 and 49.20 µg/mL) and chloroquine-sensitive K67 (IC_50_: 9.90 and 35.90 µg/mL) strains.

The findings in this report, together with all previous data, emphasize the potential of *Terminalia* species to produce secondary metabolites with potent antiplasmodial activity. Furthermore, recent reports on phytochemical studies of *Terminalia mantaly* mainly showed the presence of phenols, flavonoids, tannins, saponins, and steroids [25, 26]. Many compounds belonging to these classes of phytochemicals have been found to be highly potent against several sensitive and resistant strains of *P. falciparum* [4, 19, 27–30]. Moreover, studies on their potential mechanisms of action revealed that phenolic compounds and derivatives are very active as enzymes inhibitors. Examples of such enzymes inhibited by phenolic compounds and derivatives are aspartic proteases, xanthine oxidase, 1,5-lipoxygenase, α-glucosidase, glucose-6-phosphate dehydrogenase, carbonic anhydrase and glutathione-*S*-transferase [26, 30, 31]. It is noteworthy that nowadays some of the abovementioned enzymes including aspartic proteases, glutathione-*S*-transferase are clearly identified as potential new targets for drug discovery against malaria and several others metabolic dysfunctions of public health significance including cancer, obesity, epilepsy and gout.

## Conclusion

Results from this study clearly demonstrate the activity of *Terminalia mantaly* and *Terminalia superba* plants extracts against *P. falciparum,* the causative agent of malaria. To a reasonable extent, they also partly support the traditional uses of these plants in ethno-medicine to treat malaria. However, full validation of this use will depend on the results of detailed toxicological studies of the active extracts. The findings reported here have great scientific significance as they highlight for the first time the antiplasmodial activity of *Terminalia mantaly*. Identified extracts and fractions with very good antiplasmodial potential and selectivity will be further fractionated following activity-guided approach, and the isolated hit compounds polished and progressed towards novel anti-malarial drugs development.

## Abbreviations

CC_50_: 50% cell cytotoxic concentration
Chl: chloroform
DMEM: Dulbecco’s Modified Eagle’s Medium
DMSO: dimethyl sulfoxide
EA: ethyl acetate
EDTA: ethylene diamine tetra acetate
H: hexane
HEK239T: human embryonic kidney cells 239T
HEPES: 2-[4-(2 hydroxyethyl)piperazin-1-yl]ethanesulfonic acid
HNC: Herbier National du Cameroun
IC_50_: 50% inhibitory concentration
IC_50_*Pf*3D7: 50% inhibitory concentration on *Plasmodium falciparum* 3D7 strain
IC_50_*Pf*INDO: 50% inhibitory concentration on *Plasmodium falciparum* INDO strain
IMPM: Institute for Medical Research and Medicinal Plants Studies
l: leaf
m: methanol
mL: milliliter
mM: millimolar
MTT: 3-(4,5-dimethylthiazol-2-yl)-2,5-diphenyltetrazolium bromide
*Pf*3D7: *Plasmodium falciparum* 3D7 strain
*Pf*INDO: *Plasmodium falciparum* INDO strain
r: root
R3: final residue
RI: resistance index
RPMI: Roswell Park Memorial Institute Medium
sb: stem bark
SD: standard deviation
SI: selectivity index
SI*Pf*3D7: selectivity index on *Plasmodium falciparum* 3D7 strain
SI*Pf*INDO: selectivity index on *Plasmodium falciparum* INDO strain
*Tm*l^m^: *Terminalia mantaly* leaf methanol extract
*Tm*l^w^: *Terminalia mantaly* leaf water extract
*Tm*l^w^Chl: *Terminalia mantaly* leaf water extract chloroform fraction
*Tm*l^w^EA: *Terminalia mantaly* leaf water extract ethyl acetate fraction
*Tm*l^w^H: *Terminalia mantaly* leaf water extract hexane fraction
*Tm*l^w^R3: *Terminalia mantaly* leaf water extract final residue
*Tm*r^m^: *Terminalia mantaly* root methanol extract
*Tm*r^w^: *Terminalia mantaly* root water extract
*Tm*sb^m^: *Terminalia mantaly* stem bark methanol extract
*Tm*sb^w^: *Terminalia mantaly* stem bark water extract
*Tm*sb^w^Chl: *Terminalia mantaly* stem bark water extract chloroform fraction
*Tm*sb^w^EA: *Terminalia mantaly* stem bark water extract ethyl acetate fraction
*Tm*sb^w^H: *Terminalia mantaly* stem bark water extract hexane fraction
*Tm*sb^w^R3: *Terminalia mantaly* stem bark water extract final residue
*Ts*l^m^: *Terminalia superba* leaf methanol extract
*Ts*l^w^: *Terminalia superba* leaf water extract
*Ts*l^w^Chl: *Terminalia superba* leaf water extract chloroform fraction
*Ts*l^w^EA: *Terminalia superba* leaf water extract ethyl acetate fraction
*Ts*l^w^H: *Terminalia superba* leaf water extract hexane fraction
*Ts*l^w^R3: *Terminalia superba* leaf water extract final residue
*Ts*r^m^: *Terminalia superba* root methanol extract
*Ts*r^m^Chl: *Terminalia superba* root methanol extract chloroform fraction
*Ts*r^m^EA: *Terminalia superba* root methanol extract ethyl acetate fraction
*Ts*r^m^H: *Terminalia superba* root methanol extract hexane fraction
*Ts*r^m^R3: *Terminalia superba* root methanol extract final residue
*Ts*r^w^: *Terminalia superba* root water extract
*Ts*sb^m^: *Terminalia superba* stem bark methanol extract
*Ts*sb^w^: *Terminalia superba* stem bark water extract
w: water
